# Supplementary material for: Inbreeding parents should invest more resources in fewer offspring
Source: Proc Biol Sci. 2016 Nov 30;283(1843):20161845. doi: 10.1098/rspb.2016.1845 (PMC5136589; doi:10.1098/rspb.2016.1845)
Supplement: Supporting Information S1 [file rspb20161845supp1.pdf]

Inbreeding parents should invest more resources in fewer offspring

Supplementary Material

A. Bradley Duthie[1,*], Aline M. Lee[1], and Jane M. Reid[1],

[1] Institute of Biological and Environmental Sciences, School of Biological Sciences, Zoology Building, Tillydrone Avenue, University of Aberdeen, Aberdeen AB24 2TZ, United Kingdom [*] E-mail: aduthie@abdn.ac.uk

Manuscript elements: Figure 1, Figure 2, Figure 3, Figure 4, Figure 5, Appendix 1, Appendix 2, Appendix 3, Supplementary Material

Key Words: Inbreeding, parental investment, mate choice, reproductive strategy, relatedness, inclusive fitness

Parameter	Description
M	Female's total investment budget
m	Female's investment per offspring
m^*	Optimal female investment per offspring
n	Total number of offspring produced by a female
ζ_{off}	Number of identical-by-descent copies of a female's alleles inherited per viable offspring, scaled relative to the female's kinship to herself
r	Relatedness between a focal female and the sire of her offspring
k	Kinship between a focal female and the sire of her offspring
f	Degree to which a focal female is inbred (coefficient of inbreeding)
m_{min}	Minimum parental investment required for offspring viability
β	Inbreeding depression in offspring viability that can be mitigated by parental investment
c	Curve relating parental investment to offspring viability
γ	Focal female's inclusive fitness (i.e., the rate at which a focal female's identical-by-descent alleles are inherited by viable offspring)
γ^*	γ given optimal parental investment
m_0^*	Optimal PI for strictly monogamous parents that outbreed
m_r^*	Optimal PI for strictly monogamous parents that inbreed to the degree r

Table 1: Definitions of key parameters.

Inbreeding depression that cannot be mitigated by PI

Suppose we assume that some amount of reduction in offspring viability (ID) due to inbreeding cannot be mitigated by PI, $(1 - \Delta r)$, such that offspring viability decreases linearly with increasing Δ and r regardless of m. In this case, ζ_{off} is simply,

$$\zeta_{\text{off}} = \frac{1}{2}\left(1 + r\right)\left(1 - e^{-c\left(m - m_{min} - \beta r\right)}\right)\left(1 - \Delta r\right). \tag{S-1}$$

Note that offspring viability now decreases with Δ, but the additional strength of ID Δ is (by definition) independent of m. A simple example illustrates how ζ_{off} and therefore γ^{*} decreases with increasing Δ, while m^{*} remains unaffected. We return to the example in which $c = 1$, $m_{min} = 1$, $\beta = 1$, and $r = 1/2$ (inbreeding between first order relatives).

Figure S1 compares $\Delta = 0$ (solid curve) with $\Delta = 1/2$ (dashed curve). When strong ID exists with an effect that is independent of m, optimal PI does not change, and $m^{*} = 2.847$. Yet while m^{*} is unaffected by Δ, the additional source of ID has a predictable negative effect on ζ_{off}, with inclusive fitness $\gamma^{*} = 0.195$ (solid line) when $\Delta = 0$ and $\gamma^{*} = 0.146$ when $\Delta = 1/2$ (dashed line). The inclusion of Δ effectively relaxes the assumption that sufficient PI can always compensate for being inbred. For inbred offspring, ζ_{off} approaches $1/2(1 + r)(1 - \Delta r)$ as $m \rightarrow \infty$. As such, when $r = 1/2$ and $\Delta = 1/2$, the maximum possible ζ_{off} is 0.5625 (dashed curve) instead of 0.75 (solid curve). While this has no effect on optimal PI given that inbreeding has occurred, the existence of ID that cannot be mitigated with PI might strongly affect the inclusive fitness consequences of inbreeding versus avoiding inbreeding (e.g., $\gamma^{*}_{r=1/2}$ versus $\gamma^{*}_{r=0}$).

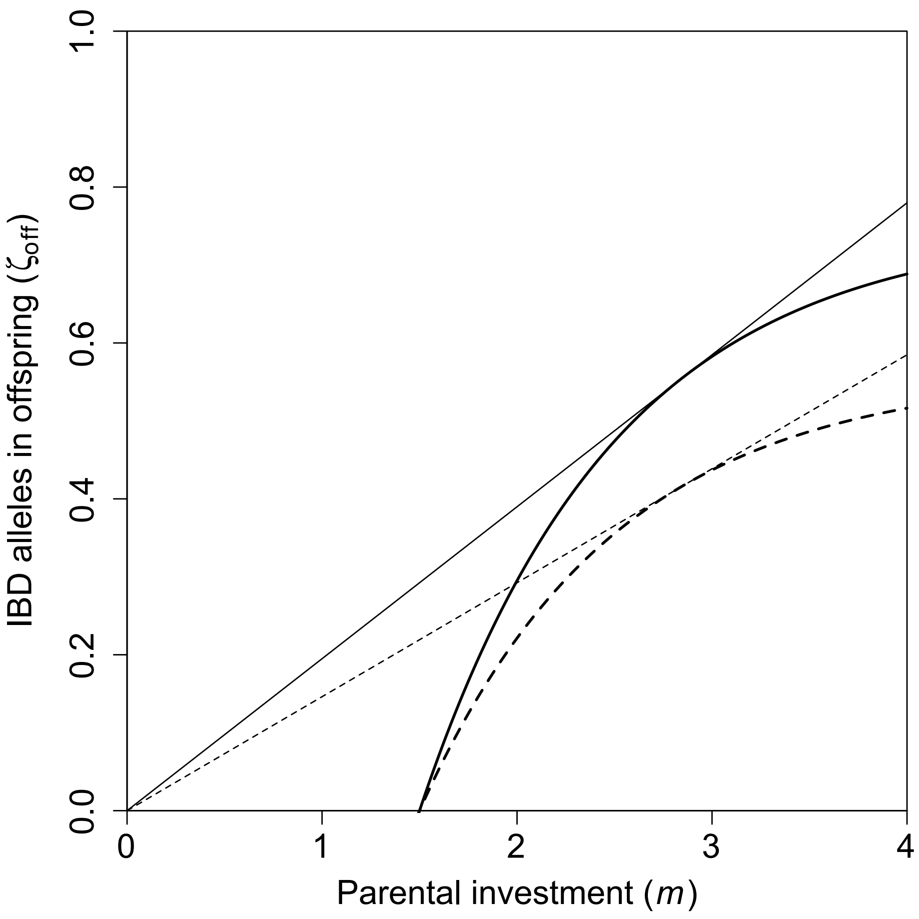

Figure S1: Relationship between parental investment (PI) and the number of a focal female's identical-by-descent alleles that are are inherited by its offspring in the absence (solid curve) and presence (dashed curve) of inbreeding depression (ID) that cannot be mitigated by increasing PI. In both curves, females inbreed with first order relatives ($r = 1/2$), the minimum amount of investment for offspring viability is one ($m_{min} = 1$), the curve of PI with offspring viability is identical ($c = 1$), and there is a small amount of ID that can be mitigated by PI ($\beta = 1$). For the solid curve, these parameters are identical to those of the dashed line in Figure 1A of the main text. However, in the dashed curve here, some additional ID ($\Delta = 1/2$) cannot be mitigated by PI, leading to lower offspring viability (compare the slope of the straight solid line with the slope of the straight dotted line, each of which passes through the origin and lies tangent to $\zeta_{\text{off}}(m)$ curves and defines optimal female inclusive fitness γ^{*}). Despite unavoidably lower offspring viability (ζ_{off}), and therefore lower optimal parent inclusive fitness (γ^{*}), optimal PI (m^{*}) does not change, as indicated by the point on the x-axis ($m^{*} = 2.847$) where straight lines are tangent to $\zeta_{\text{off}}(m)$ curves.

Consistency with biparental inbreeding models

To show that a female that inbreeds with a first order relative ($r = 1/2$) has a higher fitness than a female that outbreeds ($r = 0$) given $m_{min} = 1$, $\beta = 1$, and $c = 1$ at optimal values of $m^{*}_{r=1/2}$ and $m^{*}_{r=0}$, we define δ_r as follows,

$$\delta_r = e^{-c(m^{*}_{r} - m_{min} - \beta r)}. \tag{S-2}$$

Existing biparental inbreeding models [e.g., 1–3] implicitly assume that $\delta_{r=0} = 0$ for outbred offspring, but this is not valid in our model because $\delta_{r=0}$ will also depend on PI. However, by defining δ_r to explicitly include PI, we can thereby define inclusive fitness using the same mathematical framework as existing biparental inbreeding models, where inclusive fitness resulting from inbreeding to any degree r is determined by,

$$W_r = \frac{n}{2}\left(1 + r\right)\left(1 - \delta_r\right). \tag{S-3}$$

The right hand side of the above equation defines inclusive fitness resulting from inbreeding as appears in inequality 1 of Duthie and Reid [1], where the inequality compares inclusive fitness accrued from inbreeding with a relative r versus inclusive fitness accrued from outbreeding. Equivalent definitions of W_r also appear in Parker [2], and in Kokko and Ots [3]. By definition, $n = M/m$, so n is the total number of offspring a female produces. Existing biparental inbreeding models assume that n is constant, but n scales linearly with m in our model because females that invest more in each offspring (high m) will produce fewer total offspring (low n). To account for this, we can simply substitute M/m for n,

$$W_r = \frac{M}{2m}\left(1 + r\right)\left(1 - \delta_r\right). \tag{S-4}$$

Inclusive fitness given $r = 0$ and $r = 1/2$, and $m^*_{r=0}$ and $m^*_{r=1/2}$, can be determined by substituting some constant value for M (here for simplicity, assume $M = 1$), as the magnitude of n will not affect relative inclusive fitness.

To show that inbreeding with first order relatives returns a higher inclusive fitness than outbreeding given the above conditions assumed in our model, we can use the above equation directly to compare inclusive fitness given both $r = 1/2$ and $r = 0$, noting that $\delta_{r=1/2} = 0.26$ and $\delta_{r=0} = 0.32$ (note that $\delta_{r=1/2} < \delta_{r=0}$ because an inbreeding female invests more in her offspring, $m^*_{r=1/2} = 2.847$ versus $m^*_{r=0} = 2.146$). Consequently, we can use the above equation to show that the inclusive fitness gain of an optimally investing female that inbreeds with a first order relative is 0.195, compared with 0.159 for the outbreeding female; these values are identical to our earlier calculated values of $\gamma^*_{r=1/2}$ and $\gamma^*_{r=0}$.

Fitness (γ) across PI (m) given $\beta = 1$ & $\beta = 3$

Figure S2 shows how γ changes as a function of m, and graphically identifies m^* where γ is maximised (i.e., $\gamma = \gamma^*$). Values of m^* are identified by grey dots where γ is at its peak for four r values. Figure S2A identifies m^* where β is sufficiently low to mean that inbreeding is beneficial (γ peaks at higher values when r is higher), and Fig. S2B identifies m^* where β is sufficiently high to mean that outbreeding is beneficial (γ peaks at higher values when r is lower). As in Fig. 1B, the figure below shows that m^* always increases with increasing r and β. Consequently, inbreeding parents might often be expected to invest more in fewer offspring, relative to outbreeding parents.

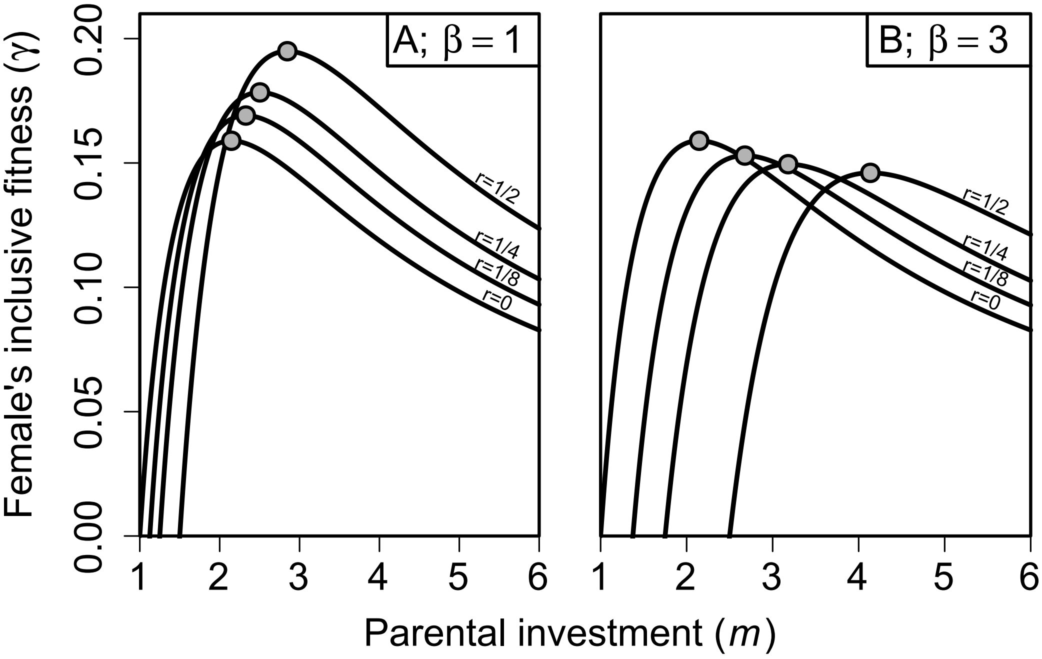

Figure S2: A focal female's inclusive fitness (γ), defined as the rate at which she increases the number of identical-by-descent alleles copies carried in her offspring, given different magnitudes of parental investment (PI). Lines show outbreeding ($r = 0$), inbreeding between outbred first cousins ($r = 1/8$), half-siblings ($r = 1/4$), and full-siblings ($r = 1/2$). Grey dots identify optimal PI (m^*) for each degree of inbreeding given magnitudes of inbreeding depression in which inbreeding (A; $\beta = 1$) and outbreeding (B; $\beta = 3$) increase parent inclusive fitness.

Fitness consequences of sub-optimal PI

One goal of theory is to provide unified explanations for otherwise disparate natural phenomena, and to thereby generate broadly applicable predictions that follow from a minimal set of general assumptions. We used inclusive fitness theory as a framework to unify previous theory developed for understanding adaptation in the context of inbreeding [e.g., 2, 4] and parental investment [e.g., 5, 6], and to thereby make general predictions for how natural selection should act across all sexual species when PI can vary with inbreeding. Our interest was therefore to broadly understand the general effect of natural selection with respect to inbreeding and PI, not to predict specific inclusive fitness consequences for different magnitudes of inbreeding or PI provided by individual parents. Nevertheless, by predicting the inclusive fitness consequences for females that inbreed and allocate PI sub-optimally (i.e., $m < m^{*}$), it is possible to gain some additional insight into how selection might affect female fitnesses in real populations.

Here we focus on the specific inclusive fitness consequences of females that inbreed and provide PI to different degrees. We consider outbred females that breed with mates of four different degrees of relatedness: non-relatives ($r = 0$), first cousins ($r = 1/8$), half siblings ($r = 1/4$), and full siblings ($r = 1/2$). We consider a focal female that inbreeds to each degree r, and allocates PI at the optimum of her own, and all other, optimal PIs (i.e., $m^{*}_{r=0}$, $m^{*}_{r=1/8}$, $m^{*}_{r=1/4}$, and $m^{*}_{r=1/2}$ for all degrees of inbreeding r). Figure S3 shows the consequences across all possible combinations of inbreeding and PI given female-only PI (left-hand column of panels) and strict monogamy (and hence biparental investment, right-hand column of panels). A focal female's inclusive fitness (γ) is shown across a range of magnitudes of ID (β) assuming that females allocate PI at $m^{*}_{r=0}$ (Fig. S3A,B), $m^{*}_{r=1/8}$ (Fig. S3C,D), $m^{*}_{r=1/4}$ (Fig. S3E,F), $m^{*}_{r=1/2}$ (Fig. S3G,H). Line weights show different degrees of inbreeding (r) from decreasing to increasing such that the thinnest lines show $r = 0$, medium thin lines show $r = 1/8$, medium thick lines show $r = 1/4$, and

the thickest lines show $r = 1/2$. Overall, Figure S3 thereby shows the inclusive fitness consequences (γ) across multiple degrees of inbreeding, PI (m), and magnitudes of ID (β).

Given female-only PI (left-hand column of panels), inclusive fitness always decreases with increasing β, except when $r = 0$ and outbreeders invest optimally (Fig. S3A), in which case inclusive fitness remains unchanged despite increasing β. As outbreeding females invest at the optimum of increasingly high inbreeding (panel rows top to bottom), female inclusive fitness decreases more rapidly with increasing β because outbreeding females are allocating PI further away from their optimum and more toward the optimum of females that inbreed (compare Fig. S3 with Fig. S2, where γ for $r = 0$ decreases along the curve away from its maximum given m^* for increasing $r = 1/8$ to $r = 1/2$). When $r > 0$, γ always decreases with increasing β, but the slope of this decrease is weakest when a female is investing at its optimum PI. For example, when $r = 1/8$, the slope of γ as a function of β is least negative in Fig. S3C, where the female is investing at its optimum $m^*_{r=1/8}$, and more negative for all other m^* (Fig. S3A,E,G). For the highest $m^*_{r=1/2}$ (Fig. S3G), $r = 1/2$ consistently has the highest γ, with a monotonic decrease in γ with decreasing r, and as females therefore invest further away from their own optimum PI (it is relevant to note that lines predicting γ in Fig. S3G would cross at higher β values, as they do at increasingly higher β in Fig. S3A,C, and E). Overall, inclusive fitness decreases with increasing β, and as a female invests further away from its optimal PI.

Given strict monogamy (and hence biparental investment, right-hand column of panels), inclusive fitness always decreases with increasing β, except again when outbreeders invest optimally (Fig. S3B). In contrast to female-only PI, given strict monogamy, parents that inbreed ($r > 0$) never have a higher inclusive fitness than parents that avoid inbreeding ($r = 0$). In fact, across all optimal PI, parent inclusive fitness always decreases

with increasing inbreeding. For example, in Fig. S3F, a focal parent that inbreeds with its half-sibling (second thinnest line) is investing optimally while all other parents do not, yet its inclusive fitness is always lower than parents that outbreed or inbreed with first cousins ($r = 0$ and $r = 1/8$, respectively), and always higher than parents that inbreed with full siblings ($r = 1/2$). This illustrates the importance of considering the inclusive fitness consequences of inbreeding in light of opportunity costs [7], such as the complete opportunity cost imposed by strict monogamy. If opportunity costs of male mating are high, then inbreeding will decrease inclusive fitness regardless of PI, as is consistent with the key conceptual point highlighted by Waser et al. [7].

Overall, Figure S3 thereby shows the inclusive fitness consequences of varying inbreeding and PI across a wide range of values for inbreeding depression. These specific cases affecting inclusive fitness highlight our general conclusion that individuals will benefit by increasing their PI at higher magnitudes of inbreeding and inbreeding depression, as illustrated by the inclusive fitness consequences of deviations from optimal PI derived in the main text.

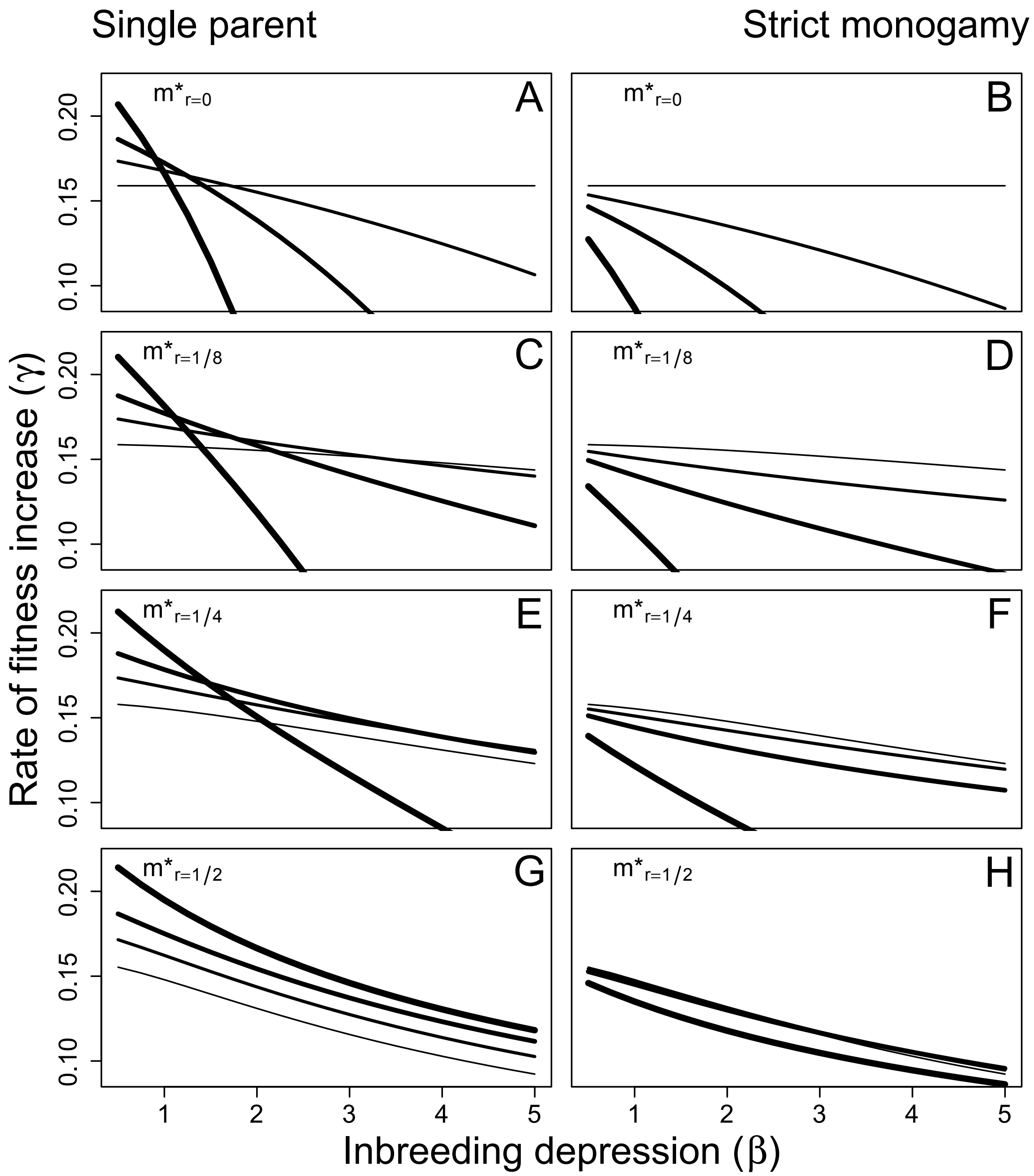

Figure S3: Relationship between the magnitude of inbreeding depression and a focal parents's inclusive fitness given optimum parental investment (m^*) for four degrees of relatedness between a focal parent and its mate ($r = \{0, 1/8, 1/4, 1/2\}$, rows top to bottom) and single parent investment (left column) versus strict monogamy (right column). Each panel therefore shows a unique combination of optimum parental investment given either single parent investment or strict monogamy. Within panels, parent inclusive fitness is plotted as a function of inbreeding depression for all combinations of r: $r = 0$ (thin line), $r = 1/8$ (medium thin line), $r = 1/4$ (medium thick line) and $r = 1/2$ (thick line).

References

[1] Duthie, A. B. & Reid, J. M., 2015 What happens after inbreeding avoidance? Inbreeding by rejected relatives and the inclusive fitness benefit of inbreeding avoidance. *PLoS One* **10**, e0125140, doi:10.1371/journal.pone.0125140.

[2] Parker, G. A., 2006 Sexual conflict over mating and fertilization: an overview. *Philosophical Transactions of the Royal Society B* **361**, 235–259, doi:10.1098/rstb.2005.1785.

[3] Kokko, H. & Ots, I., 2006 When not to avoid inbreeding. *Evolution* **60**, 467–475.

[4] Parker, G. A., 1979 Sexual selection and sexual conflict, in *Sexual selection and reproductive competition in insects* (eds. M. S. Blum & N. A. Blum), pp. 123–166, New York: Academic Press, Inc.

[5] Macnair, M. R. & Parker, G. A., 1978 Models of parent-offspring conflict. II. Promiscuity. *Animal Behaviour* **26**, 111–122, doi:10.1016/0003-3472(78)90010-6.

[6] Parker, G. A. & Macnair, M. R., 1978 Models of parent-offspring conflict. I. Monogamy. *Animal Behaviour* **26**, 97–110, doi:10.1016/0003-3472(78)90009-X.

[7] Waser, P. M., Austad, S. N. & Keane, B., 1986 When should animals tolerate inbreeding? *American Naturalist* **128**, 529–537.
